# Programming Tactic Behaviors of Active Colloids via Surface Charge

**DOI:** 10.1021/acsnano.5c02441

**Published:** 2025-06-07

**Authors:** Zuyao Xiao, Priyanka Sharan, Juliane Simmchen

**Affiliations:** † Department of Physical Chemistry, Technische Universität Dresden, Dresden 01069, Germany; ‡ Pure and Applied Chemistry, University of Strathclyde, Glasgow G11XL, U.K.

**Keywords:** active colloids, self-diffusiophoresis, phoretic mobility, surface functionalization, chemotaxis, rheotaxis, microfluidics

## Abstract

Self-diffusiophoretic colloids move autonomously by creating chemical gradients. These self-generated gradients lead to fluid motion at the particle surface, also known as phoretic slip, which determines the direction of colloidal motion. Phoretic slip is often modeled as dependent on the local chemical gradient and a material-dependent property called phoretic mobility. Reversing the direction of propulsion would require fine-tuning of these parameters. In this work, we show that by changing the charge of the colloids which has an effect on the phoretic mobility, we can indeed achieve a reversal in the direction of propulsion of Janus micromotors. In addition, we report that by simply tuning the surface charges of colloids, we can achieve reversals in much more complex tactic behaviors such as chemotaxis and rheotaxis. This work demonstrates a method for creating programmable active colloids by subtly tuning surface properties, contributing to the understanding of active matter.

## Graphical abstract

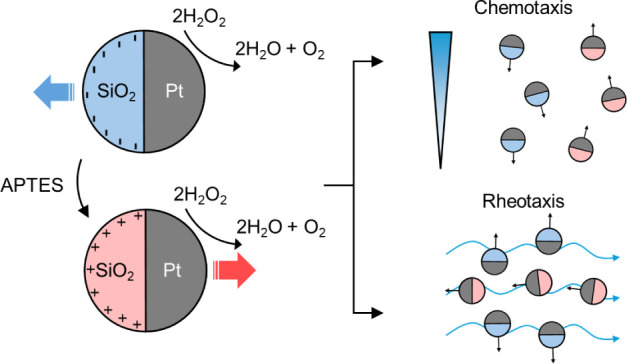

## Introduction

Microorganisms often adapt their motion in response to environmental cues, exhibiting behaviors such as chemotaxis (movement toward or away from chemical gradients) and rheotaxis (alignment and swimming relative to fluid flows).^1–3^ For example, E. coli adjusts its run-and-tumble pattern to navigate through nutrient gradients,^4^ sperm cells rely on chemical attractants to locate the ovum,^5^ and some planktonic species orient against currents to enhance nutrient uptake.^6^ Inspired by these biological systems, synthetic active colloids have been developed to mimic such tactic responses in engineered settings.^7–13^ These colloidal swimmers hold significant promise for applications in targeted drug delivery,^14^ environmental remediation,^15^ and microscale assembly.^16^

Despite these advancements, precise understanding—which proceeds control over the tactic behaviors of synthetic active colloids, especially in chemically driven systems—remains challenging.^17^ Current strategies often rely on external fields, such as magnetic,^18^ electric,^19^ or acoustic stimuli,^20^ to steer particle motion. While effective in certain contexts, these approaches can be impractical when the propulsion mechanism is intrinsically chemical or when large-scale external fields are undesirable. Modulating tactic behaviors purely through the intrinsic properties of chemically propelled particles presents a promising yet underexplored alternative.

In this work, we address this challenge by modifying the surface properties of platinum-coated silica (Pt@SiO_2_) Janus particles to control their interactions with surrounding media and tune their tactic behaviors. Specifically, we adjust the surface charge of the silica (SiO_2_) hemisphere to influence particle navigation in chemical gradients and fluid flows. This functionalization-based approach enriches the fundamental understanding of active matter and offers a versatile method for fine-tuning the motion of chemically driven colloids in diverse environments.

## Results and Discussion

### Modulation of Self-diffusiophoresis

Self-diffusiophoresis refers to the autonomous motion of a colloidal particle driven by an asymmetric solute concentration-a gradient that the particle itself generates or sustains through surface chemical reactions. These localized reactions establish solute concentration gradients near the particle’s surface, with gradients running tangentially to the particle-fluid interface. These tangential gradients create interactions between the solute and the particle surface, inducing a thermodynamic force that drives fluid flow near the interface.^21^

In the general expression of self-diffusiophoresis, the apparent slip velocity *V*_*s*_ at the particle surface is expressed as

1 $$V_{s}=\mu \nabla C$$

where μ represents the phoretic mobility depending on the specific interactions between the solute and the particle-fluid interface and ∇*C* denotes the tangential component of the solute concentration gradient.^21^ Although this model is remarkably simple, it has been regarded as capturing the essential physics of self-diffusiophoresis and is widely used.^22,23^ It is important to note that the phoretic mobility μ is treated as a system parameter; it is generally unknown a priori and is determined by fitting the model to experimental observations.^24^ A more detailed discussion of these parameters and their determination will be presented in a later section of this paper.

Self-diffusiophoresis theory highlights two primary strategies to reverse or reorient the slip flow driving the particle. The first involves modulating the chemical gradient ∇*C*, achieved by varying the reactant concentration,^25–27^ altering the catalytic activity,^28,29^ or changing particle geometry.^30,31^ These adjustments redistribute reaction products and solutes, thus steering the particle’s motion. The second, less explored method involves altering the particle’s phoretic mobility μ. This can be achieved by tuning surface properties such as surface charge^32^ or hydrophilicity^33^ to modify the interaction between the solutes and the particle surface, thereby altering the phoretic mobility, the resulting flow, and ultimately the particle motion, as illustrated in Figure 1. Experimentally, we attempt that by functionalizing a negatively charged SiO_2_ surface with an amino-silane to obtain a positively charged surface (we denote them as +SiO_2_ in the following). This changes the overall zeta potential and assumably the sign of the phoretic mobility, which is followed by an inversion of the flow and propulsion direction.

**Figure 1:**
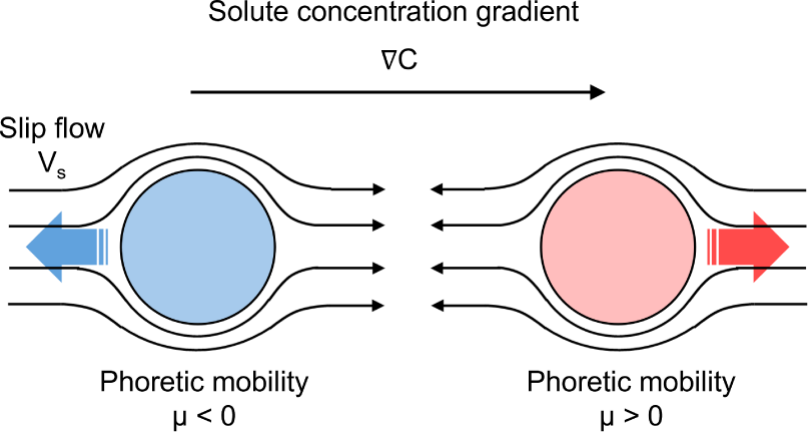
Schematic illustration of how phoretic mobility μ governs slip flow *V*_*s*_ around particles in a solute concentration gradient ∇*C*. In the same gradient, a reversal in the sign of the phoretic mobility μ leads to a reversal in the direction of the slip flow, which consequently reverses the particle's translational velocity.

### Propulsion Reversal via Surface Functionalization

In our experiments, we observed the motion of both unmodified (Pt@SiO_2_) and functionalized (Pt@+SiO_2_) Janus particles in H_2_O_2_ solutions with concentrations ranging from 0.1% to 10%. Before functionalization (Figure 2A), the SiO_2_ particles exhibited a negative surface charge because of partially dissociated hydroxyl (−OH) groups on the SiO_2_ surface, with a zeta potential of approximately −30 mV, as determined by dynamic light scattering measurements. Under these conditions, the unmodified particles were half-coated with a layer of Pt and subsequently exhibited motion directed toward the SiO_2_ side in H_2_O_2_ (Video S1), consistent with observations reported in previous studies.^34^ Surface charge modification was achieved by functionalizing the SiO_2_ spheres with 3-aminopropyltriethoxysilane (APTES) followed by Pt deposition on one-half of these spheres (Figure 2D). During the functionalization, the ethoxysilane groups of APTES formed stable Si–O–Si bonds with the surface −OH groups of the SiO_2_ hemisphere, leaving amino (−NH_2_) groups exposed. At near-neutral pH, these −NH_2_ groups become protonated (−NH_3_^+^), shifting the zeta potential to approximately +5 mV. Consequently, we observed that the APTES-functionalized Pt@+SiO_2_ moved toward the Pt hemisphere in H_2_O_2_ (Video S2).

**Figure 2:**
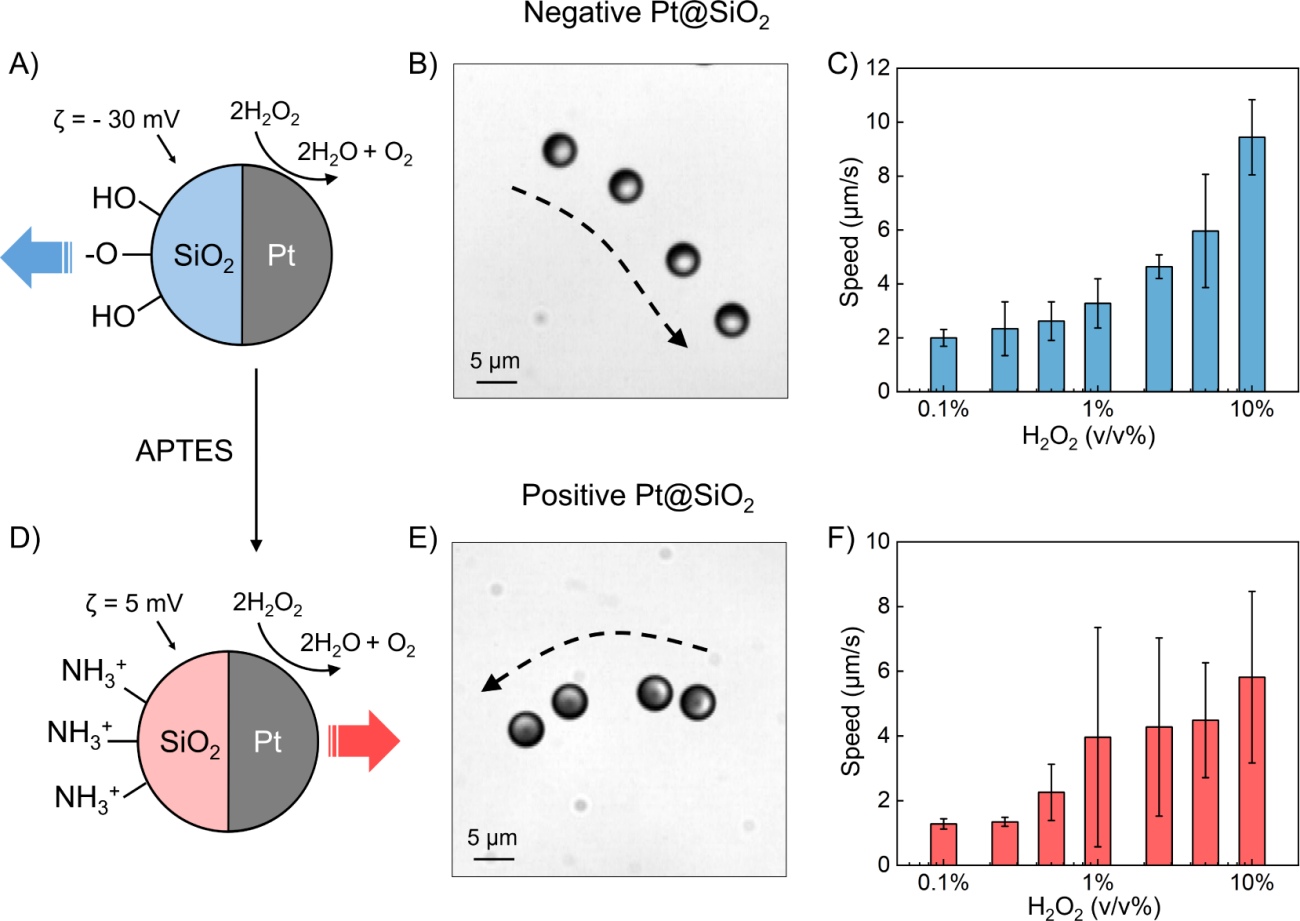
Reversal of Pt@SiO_2_ propulsion direction via surface functionalization. (A) Schematic of a Pt@SiO_2_ Janus particle with partially dissociated −OH groups on the SiO_2_ hemisphere, yielding a negative surface charge. (B) Negatively charged Pt@SiO_2_ particles swim with the inert (SiO_2_) side leading, and (C) their speed increases with higher H_2_O_2_ concentrations. (D) Functionalization with APTES grafts −NH_2_ groups onto the SiO_2_ side, creating positively charged Pt@+SiO_2_. (E) These positively charged particles now move with the catalytic (Pt) side leading, and (F) their propulsion velocity also exhibits a dependence on H_2_O_2_ concentration.

The contrasting trajectories of negatively and positively charged particles are illustrated in Figure 2B,E. Negatively charged particles (unmodified) exhibited motion with the SiO_2_ side leading, while positively charged particles (APTES-modified) moved with the Pt side leading. Figure 2C,F further demonstrate that the propulsion speeds of both particle types increased with higher H_2_O_2_ concentrations, attributed to enhanced catalytic activity at increased reactant levels. Notably, while both particle types accelerated, positively charged particles exhibited slightly lower speeds than negatively charged ones. This difference is likely due to the smaller absolute value of the positive zeta potential compared to the negative zeta potential, resulting in a weaker slip flow. However, as the primary focus of this study is on motion direction rather than activity, this aspect was not explored further.

To understand the mechanism underlying the reversal of propulsion, it is essential to examine how modifications to the particle surface affect the phoretic mobility. Since the sole alteration in the system is the particle’s surface property, this change effectively influences the phoretic mobility. Recall that the phoretic mobility depends on the specific interactions between the solute and the particle surface:

If the solute is assumed to be neutral, such as the O_2_ produced by the Pt-catalyzed decomposition of H_2_O_2_ (a process widely discussed in the context of neutral self-diffusiophoresis),^22,35–37^ the phoretic mobility can be expressed as $\mu =\frac{k_{B}T}{\eta }KL^{*}$ , where *k*_*B*_ is the Boltzmann constant, *T* is the temperature, η is the fluid viscosity, and KL* is the interaction parameter. The sign of KL* denotes whether the solute is attracted to (KL* > 0) or repelled from (KL* < 0) the particle surface.^21^ Some theoretical models assume that oxygen interacts repulsively (KL*) with a bare SiO_2_ surface in order to remain consistent with experimental observations.^22^ However, direct experimental evidence regarding the exact nature of these interactions-whether van der Waals force, hydrophobic force, or excluded volume force-is still lacking.^17^ In line with previous results, one may assume repulsive interactions for unmodified SiO_2_. After functionalization via APTES, since the precise modification of all underlying interactions cannot be rigorously justified, it is plausible to assume that the interaction becomes attractive, as suggested by the observed reversal in the propulsion direction.

If the solute species are charged, the underlying mechanism remains similar to that of neutral self-diffusiophoresis, but the charged ions drive the fluid flow now and propel the particle via ionic-diffusiophoresis.^23,27,38,39^ It has been reported that Pt@SiO_2_ can be propelled by ionic diffusiophoresis, driven by charged intermediates produced during the electrochemical decomposition of H_2_O_2_.^25,40^ In ionic diffusiophoresis, when considering only the contributions from the charged species, the phoretic mobility can be approximately expressed as $\mu =\frac{k_{B}T}{\eta }\frac{\epsilon \zeta }{e}\beta$ , where ϵ is the dielectric permittivity of the medium, ζ is the zeta potential of the particle, *e* is the elementary charge, β is the ion diffusivity difference factor and defined as $\beta =\frac{D_{+}-D_{-}}{D_{+}+D_{-}}$ , with *D*_+_ and *D*_–_ being the diffusivities of the cation and anion, respectively.^23^ In contrast to neutral diffusiophoresis, the interactions governing ionic diffusiophoresis are well understood theoretically since Coulomb interactions are both long-ranged and straightforward. For simplicity, we assume the decomposition of H_2_O_2_ as H_2_O_2_ = 2H^+^ + 2OH^–^ + O_2_.^41^ Due to the difference in ionic diffusivities (β = 0.27), the diffusion of ions from the Pt cap generates an electric field. When the particle surface is negatively charged (ζ < 0), this ion-induced electric field drives fluid flow toward the Pt cap, thereby propelling the particle in the direction of the SiO_2_ side. Conversely, when the surface is rendered positively charged after APTES functionalization, the direction of the induced flow reverses and the propulsion is directed toward the Pt cap.

Based on these two plausible mechanisms we proposed—neutral versus ionic diffusiophoresis—COMSOL simulations were performed (see results in Figures S2 and S3). Both sets of simulations confirm that a reversal in the phoretic mobility results in a corresponding reversal of the propulsion direction. Although the propulsion mechanism and hence the reversal mechanism for Pt@SiO_2_ remains a subject of ongoing debate, the experimental observation of propulsion reversal firmly supports the notion that surface functionalization fundamentally alters the interactions at the particle interface and, consequently, the phoretic mobility that drives particle motion.

### Chemotactic Behavior

To investigate the effect of surface charge reversal on chemotactic behavior, we established a static H_2_O_2_ concentration gradient within a microfluidic device as described in our previous work (see setup in Figure S4 and more details in Experimental Section).^42^ We introduced both Pt@SiO_2_ and Pt@+SiO_2_ particles into the gradient. A previous study has reported positive chemotaxis for negatively charged Pt@SiO_2_ particles; however, those experiments combined chemical gradients with background flows, making it challenging to distinguish between chemotaxis and flow-induced rheotaxis.^43^ Indeed, several independent investigations have highlighted that external flow can significantly influence particle trajectories and potentially influence or obscure chemotactic behavior.^44–47^

In our experiments, we eliminated any background flow using a stop-flow microfluidic system, ensuring that the observed motion arises solely from the imposed H_2_O_2_ gradient. A more detailed description on the concentration gradient can be found in our previous study.^42^ Under these static conditions, Pt@SiO_2_ and Pt@+SiO_2_ particles exhibited chemotaxis with directions depending on their surface charge. Negatively charged Pt@SiO_2_ particles exhibited negative chemotaxis, migrating toward lower H_2_O_2_ concentrations (Video S3). Time-lapse images (Figure 3A) confirmed the downward movement of individual negatively charged particles, and the typical trajectories of more particles (Figure 3B) showed a clear preference for motion away from the higher concentration region. This is further supported by the Rose plot in Figure 3C, indicating a strong prevalence of the moving directions toward approximately 270^◦^. In the same setup, positively charged Pt@+SiO_2_ particles displayed positive chemotaxis, moving up the gradient toward regions of higher H_2_O_2_ concentration (Video S4), supported by the microscope image and the trajectories (Figure 3D,E). The corresponding Rose plot (Figure 3F) exhibited a prominent peak around 90^◦^, illustrating the taxis in the opposite direction of negatively charged particles.

**Figure 3:**
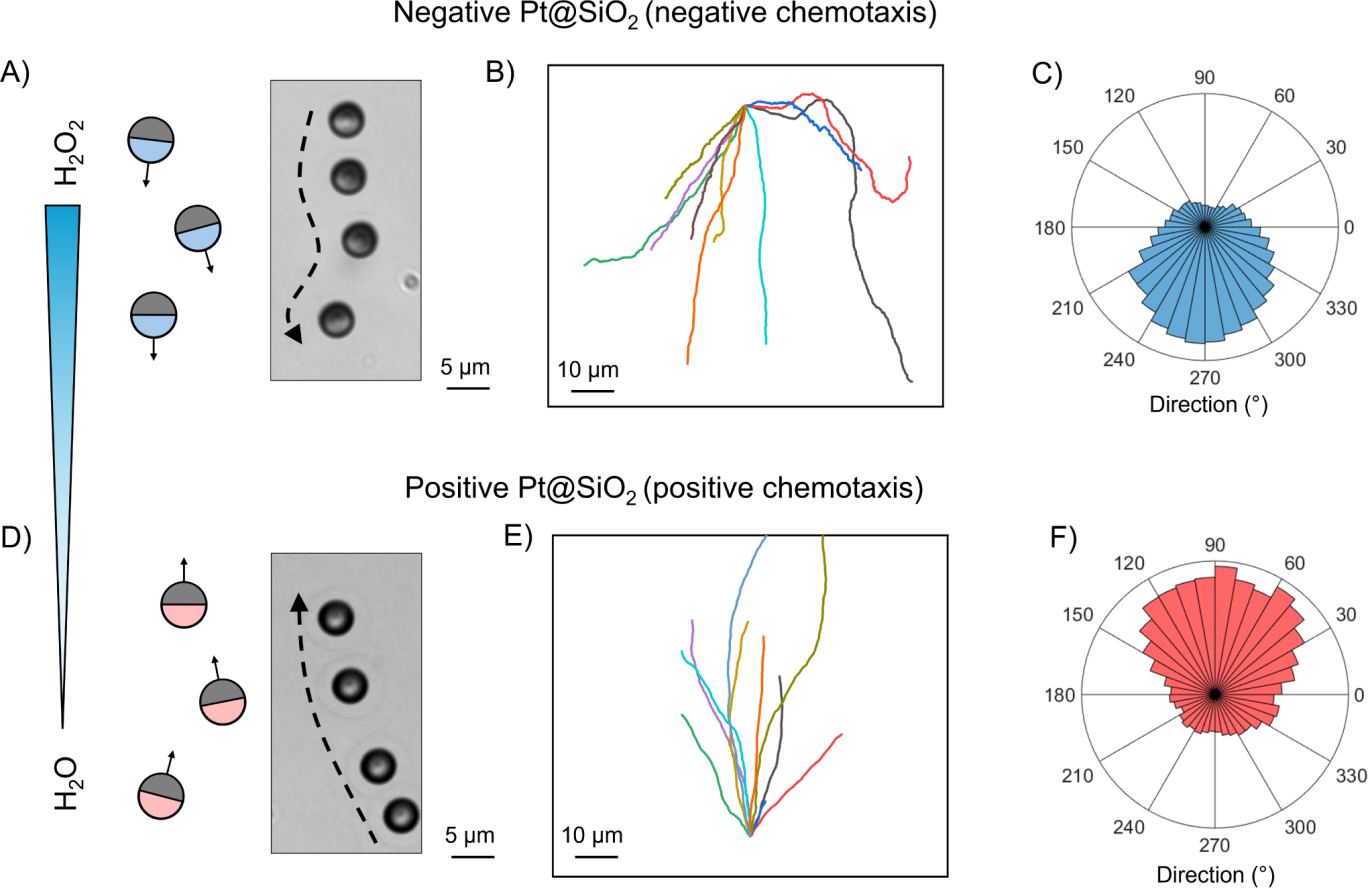
Chemotaxis of Pt@SiO_2_ particles in the H_2_O_2_ gradient. (A) Schematic illustration and time-lapse microscopy image of a negatively charged Pt@SiO_2_ particle undergoing negative chemotaxis, migrating toward lower H_2_O_2_ concentration. (B) Representative trajectories and (C) the Rose plot of negatively charged particles, showing their predominant downward motion. (D) Schematic illustration and time-lapse microscopy image of a positively charged Pt@SiO_2_ particle undergoing positive chemotaxis, migrating toward higher H_2_O_2_ concentration. (E) Representative trajectories and (F) the Rose plot of positively charged particles, showing their predominant upward motion.

According to the theoretical framework established by Popescu and coworkers,^48^ the orientation of a Janus particle in a solute gradient depends on the difference in phoretic mobility between its two hemispheres (Pt vs SiO_2_ and Pt vs +SiO_2_, respectively). This framework allows for the prediction of a Janus particle’s orientation in the chemical gradient. However, measuring individual mobilities in H_2_O_2_ solutions is challenging due to factors such as the lack of deep understanding on the phoretic mobility, the limited availability of standard protocols for mobility quantification at the microscale, and the dependence on the in situ created concentration of the reaction products.

Fitting the above-given observations into the scheme established by Popescu et al.^48^ is not straightforward, but despite their opposite migration directions, both types of particles maintained a similar orientation in the fuel concentration field, namely, the Pt cap aligned in the same way relative to the gradient and pointing toward higher H_2_O_2_ concentration. This common orientation is offset by the slip flow around the positively charged particles being reversed relative to the negatively charged ones, yielding opposite chemotactic directions. To further investigate the reorientation dynamics of particles over time, we tracked their orientation changes and calculated their mean square angular displacement (MSAD) under various conditions (Figure S6). The data indicate that both particle types exhibit comparable angular fluctuations regardless of speed range or chemotactic index. Although the MSAD of negatively charged particles is slightly higher than that of the positively charged particles.

These above results emphasize how subtle modifications to surface properties, such as charge functionalization, can drastically alter the chemotactic behavior of self-propelled colloids, which indicates that future stimuli-dependent functionalization will become an asset.

### Rheotactic Behavior

To examine the effect of surface functionalization of SiO_2_ hemisphere on rheotactic behavior, we introduced both Pt@SiO_2_ and Pt@+SiO_2_ particles into a microfluidic channel with a uniform H_2_O_2_ flow (no imposed chemical gradient). Under these conditions, the H_2_O_2_ concentration remained spatially uniform, and the particles experienced a steady flow from left to right (see setup in Figure S5 and more details in Experimental Method).

Consistent with previous studies,^45^ negatively charged Pt@SiO_2_ particles exhibited cross-stream migration under these flow conditions (Video S5). Time-lapse microscopy (Figure 4A) revealed that these particles tended to drift either upward or downward relative to the main flow axis, as depicted in the trajectories shown in Figure 4B. Rose plots (Figure 4C) of the direction of motion displayed deviation from the purely downstream direction, reflecting a lateral deflection caused by their interaction with the chemical flow field.

**Figure 4:**
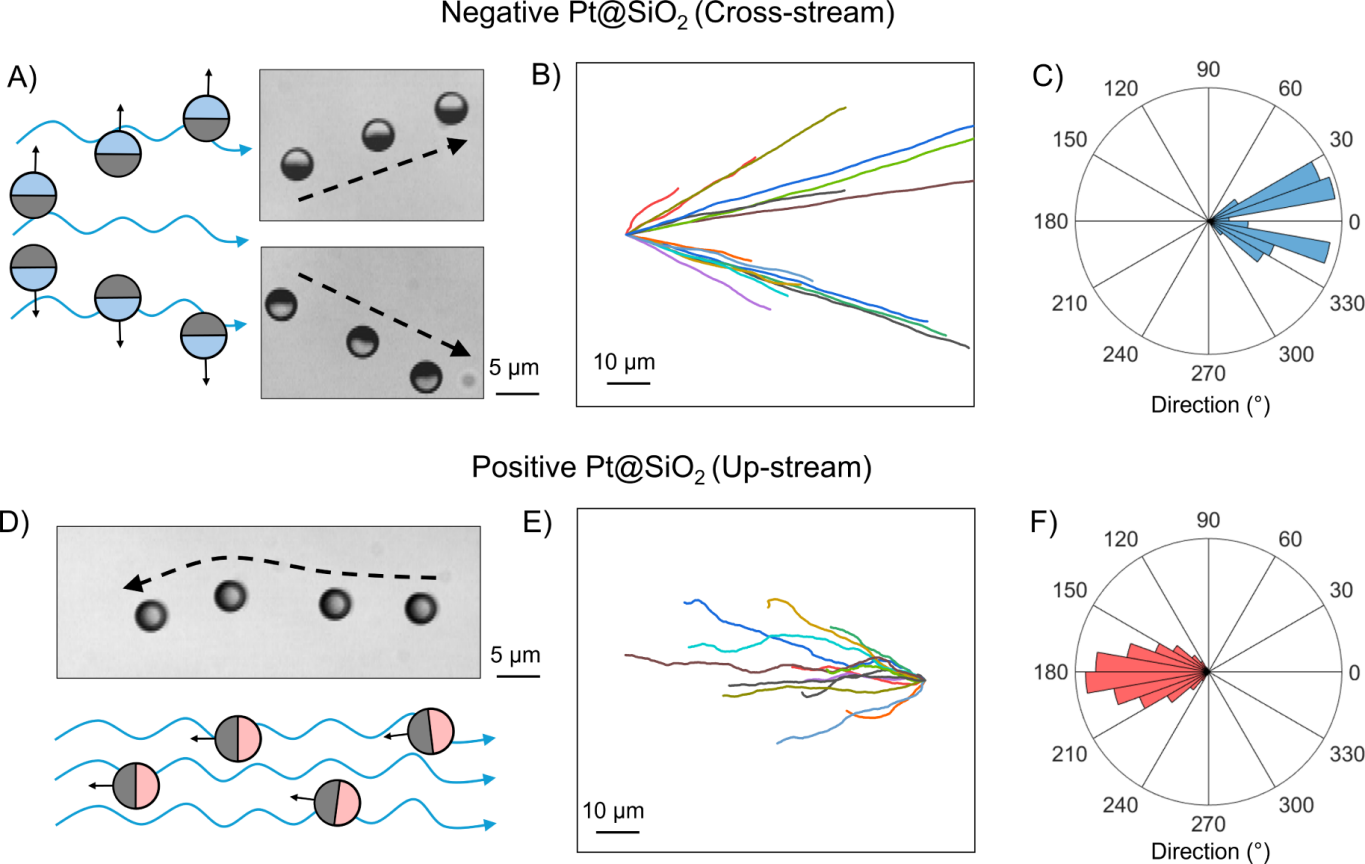
Rheotaxis of Pt@SiO_2_ particles in the H_2_O_2_ flow. (A) Schematic illustration and time-lapse microscopy image of a negatively charged Pt@SiO_2_ particle moving cross-stream in the flow. (B) Representative trajectories and (C) Rose plot of negatively charged particles, showing their lateral drift relative to the flow. (D) Schematic illustration and time-lapse microscopy image of a positively charged Pt@+SiO_2_ particle moving upstream against the flow. (E) Representative trajectories and (F) Rose plot of positively charged particles, showing their upstream motion against the flow.

In contrast, positively charged Pt@+SiO_2_ particles exhibited an entirely different rheotactic behavior. Instead of lateral drift, these particles displayed pronounced upstream migration, propelling themselves directly against the flow of H_2_O_2_ (Video S6). Time-lapse images (Figure 4D) highlighted this unique behavior: rather than being carried along with the flow or drifting sideways, the positively charged particles reoriented and actively moved upstream. Trajectories (Figure 4E) and Rose plots (Figure 4F) confirmed a strong preference for the motion of particles opposite the flow direction. We also observed that at sufficiently high flow speeds, the particles no longer maintain this upstream migration; instead, they are dragged down with the flow and periodically “jump″ off the substrate before settling back down, repeating this cycle (Figure S7 and Video S7), which is consistent with Cu@SiO_2_ particles.^49^ In the “jumping” behavior, the particles periodically get detached from the substrate and re-approaches the substrate. This results in periodic fluctuations in speed and height of the particles from the substrate which can be confirmed by the repeated focusing and de-focusing of the particles from the plane of view (Figure S8).

Notably, the propulsion modes of the particles remained consistent with those observed in static conditions. Negatively charged particles continued to move with the SiO_2_ side leading, while positively charged particles moved with the Pt side leading. However, unlike in the chemotaxis experiments, where both charge states shared a similar orientation in the gradient, their orientations here differ markedly in the fluid flow. Negatively charged particles often orient their cap sideways (either upward or downward), facilitating lateral drift, whereas positively charged particles rotate until the Pt cap faces upstream, enabling them to move against the flow. According to Katuri et al.^45^ the orientation of active particles under flow depends on multiple factors-particle density, wall slip, surface charge, and the balance between self-propulsion and external flow. Consequently, modifying the charge of the SiO_2_ surface can affect several of these parameters simultaneously, making it difficult to distinguish a single dominant mechanism behind the observed rheotactic differences. Nevertheless, the stark contrast between cross-stream migration in negatively charged particles and upstream migration in positively charged particles underscores how effectively surface charge can control Janus colloid behavior under uniform flow conditions, see overview given in Figure 5.

**Figure 5:**
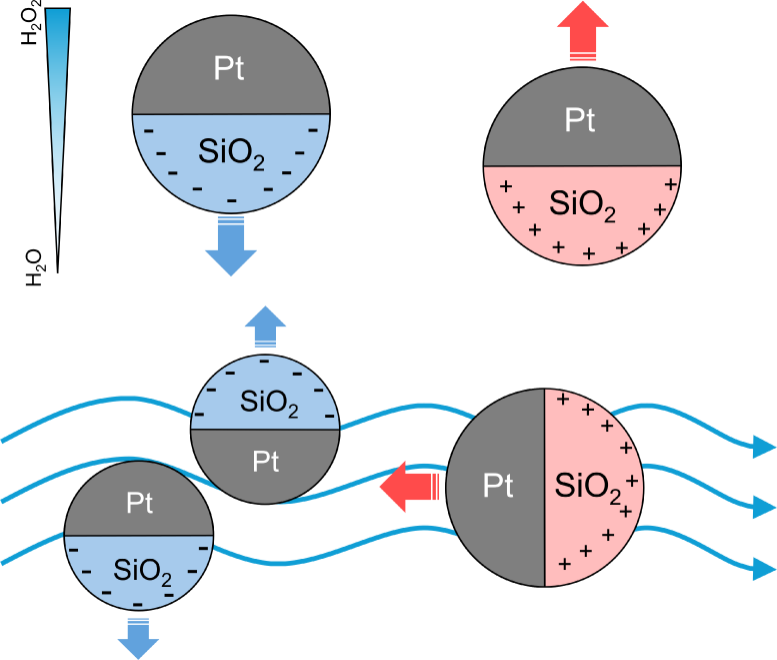
Exemplary dynamics for Pt@SiO_2_ and Pt@+SiO_2_ in complex environments.

## Conclusion

Our findings demonstrate that chemically modifying the surface of Pt@SiO_2_ Janus particles in order to control the overall particle charge, measured through the zeta potential, can profoundly influence their swimming behaviors as well as their tactic response to external stimuli such as chemical gradients and fluid flows (see schematics in Figure 5). By functionalizing the SiO_2_ hemisphere with APTES, we were able to reverse the motion direction, providing direct experimental evidence of the theoretical principles underpinning self-diffusiophoresis. Furthermore, these different swimming modes translate into different responses to both, chemical fuel gradients as well as applied external flows. Interestingly, these opposite behaviors resemble strongly what we observed for Cu@SiO_2_ particles in analogous setups.^42,49^ Given that this set of particles also showed slightly positive zeta potentials the charge, and the subsequent change of interactions between the particle surface and the products within a thin layer around the particles seem to be the determining factor influencing the surface mobilities. This is in line with Anderson’s assumptions^21^ and Popescu’s systematic predictions for chemotactic behaviors.^48^ This work highlights the potential of surface-charge-tuning as a versatile strategy for precisely modulating the motion of chemically driven colloids, opening new avenues for a broad range of advanced applications.

## Experimental Section

### Preparation of Pt@SiO_2_ and Pt@+SiO_2_ Janus Microspheres

Pt@SiO_2_ and Pt@+SiO_2_ were prepared by sputtering Pt on one-half of the SiO_2_ microspheres, respectively. Negative SiO_2_ microspheres were purchased from Sigma-Aldrich (5 wt %, 5 μm in diameter). Positive +SiO_2_ microspheres were prepared by mixing 500 μL of SiO_2_ (5 wt %, 5 μm in diameter), 1.5 mL of ammonia, 15 mL of ethanol, and 15 μL of APTES and stirring for 24 h. The prepared positive microspheres were then washed with absolute ethanol three times. Then both microspheres were suspended in ethanol (50 μL) and dispersed by ultrasound. The suspension was subsequently drop-casted on a plasma-cleaned glass slide to form a monolayer. Then, a thin layer of Pt was deposited on the monolayer using a sputtering system. Finally, the prepared Janus particles were released in DI water by sonication.

### Motion Experiments

For each experiment, 8 μL of the Pt@SiO_2_ suspension was pipetted onto a plasma-cleaned glass slide. The particles were then allowed to sediment near the slide surface, thereby reaching a relatively stable height over time. Subsequently, 2 μL of H_2_O_2_ solution at various concentrations was rapidly introduced. Particle motion was observed under an upright microscope (Zeiss Axio Observer) and recorded with a Zeiss camera at 30 fps.

### Microfluidic Setup for Chemotaxis Assays

Chemotaxis was conducted in a feedback-loop microfluidic system, following the previous procedure.^42^ The gradient was generated using a microfluidic chip (Fluidic 172, Microfluidic Chip Shop GmbH, Germany) mounted on an upright microscope. The setup comprised an OB1Mk3+ pressure controller, a MUX wire valve controller, two MFS4D flow sensors, and three 3/2 microfluidic valves (all from Elveflow, France), interconnected via PTFE tubing with an inner diameter of 794 μm. The pump and valves were regulated by Elveflow’s ESI software, which automatically adjusted flow rates through a feedback loop to compensate for any fluctuations. To establish a stable H_2_O_2_ gradient, the chip was first flushed for at least 1 min with water and 1% H_2_O_2_ via their respective inlets. The colloidal particles were then introduced through the water channel. Subsequently, the flow was stopped by applying equal pressures at the inlet and outlet channels, ensuring a quiescent environment. Under these stop-flow conditions, particles experienced a linear H_2_O_2_ gradient without turbulent disturbance.

### Microfluidic Setup for Rheotaxis Assays

Rheotaxis was investigated in a microfluidic system based on the protocol reported before.^49^ Square glass capillaries 1 mm × 1 mm cross-section, 0.2 mm wall thickness, 100 mm length; Vitrocom) were first soaked overnight in 30% H_2_O_2_ (to remove any organic residues), then rinsed thoroughly with deionized water and dried. An aqueous suspension of Pt@SiO_2_ containing 5 μm SiO_2_ tracer particles was injected into the capillary, which was mounted onto an in-house 3D-printed microscope stage. The particles were allowed to settle on the substrate before any external flow was applied. Subsequently, the capillary was connected to a microfluidic pressure pump (Flow EZ, Fluigent) via polyethylene tubing. A 2.5% H_2_O_2_ solution was pumped through the channel by manually controlling the applied pressure. The flow speed was estimated by monitoring the motion of SiO_2_ tracer particles. As a control for no-flow experiments, the same 2.5% H_2_O_2_ solution was introduced into the capillary with a pipet instead of connecting to the pump. In both cases, particle motion was recorded in real-time using an upright microscope for subsequent analysis.

### Data Analysis

The videos are processed and analyzed using MATLAB to coordinate the moving particles and calculate their instantaneous velocities and angles of motion.

## Finite Element Simulations

To simulate the self-diffusiophoresis of Pt@SiO_2_ in H_2_O_2_, we built a 2D axis-symmetric COMSOL model where one Janus particle, with 5 μm in diameter, is placed at the center of a cubic box with sides setting 100 μm. A half sphere is designated as the Pt surface, while the other is the passive SiO_2_ surface. The cube serves as the computational domain and is filled with H_2_O_2_.

### Neutral Self-diffusiophoresis

In neutral self-diffusiophoresis, two modules in COMSOL were used: Transport of Diluted Species and Creeping Flow. The concentration profile of solute (O_2_) and fluid flow within the domain are solved using a nonlinear steady-state solver implemented in COMSOL.

In the Transport of Diluted Species module, the decomposition of H_2_O_2_ to H_2_O and O_2_ occurs on the Pt surface. For small Péclet numbers (neglect convection) and at steady-state, the concentration of O_2_ is governed by the following (eq 2)­

2 J=D∇C

where *D* is the O_2_ diffusivity and *C* is the O_2_ concentration. The Janus particle in our model produces O_2_ at a flux of *J* only at the Pt surface, whereas the SiO_2_ sphere is chemically inert *J* = 0. At the edge of the calculated domain, the O_2_ concentration is set to be constantly 0.

In the creeping flow module, the fluid flow is governed by the following eqs 3 and 4:

3 ∇·u=0

4 $$\nabla p=\eta \nabla^{2}u$$

Where *p* is the pressure, η is the dynamic viscosity of water, and *u* is the fluid flow velocity.

The two modules are coupled through a neutral diffusiophoretic boundary condition on the sphere surfaces (eq 5):

5 $$V_{s}=\mu \nabla C$$

Where *V*_*s*_ is the slip velocity on the particle surface, μ is the phoretic mobility.

### Ionic Self-diffusiophoresis

In Ionic self-diffusiophoresis, three modules in COMSOL were used: Transport of Diluted Species, Electrostatic, and Creeping Flow. The concentration profile of charged species (H^+^, OH^–^), the generated electric field and fluid flow within the domain are solved.

In the Transport of Diluted Species module, the release of H^+^ and OH^–^ occurs on the Pt surface. For small Péclet numbers (neglect convection) and at steady-state, the concentration of them are governed by the following (eq 6):

6 $$J_{\pm }=-D_{\pm }\nabla C_{\pm }+\frac{eD_{\pm }}{K_{B}T}C_{\pm }E$$

where *D*_±_ is the diffusivity of ions, *C*_±_ is the concentration of ions, *e* is the elementary charge, *K*_*B*_ is the Boltzmann constant, *T* is the temperature, and *E* is the electric field with *E* = ∇ϕ . The Janus particle produces H^+^ and OH^–^ at a flux of *J*_±_ at the Pt surface, whereas the SiO_2_ sphere is chemically inert *J* = 0. At the edge of the calculated domain, the ions concentration is set to be equilibrium concentration in H_2_O.

The electric potential ϕ is governed by the Poisson (eq 7):

7 $$-\epsilon \nabla^{2}\phi =\rho_{e}=e\left(C_{+}-C_{-}\right)$$

where ϵ is the dielectric permittivity of H_2_O, ρ_*e*_ is the space charge density.

In the creeping flow module, the fluid flow is governed by the following (eqs 8 and 9):

8 ∇·u=0

9 $$\nabla p=\eta \nabla^{2}u$$

Where *p* is the pressure, η is the dynamic viscosity of water, and *u* is the fluid flow velocity.

The slip flow is calculated through a electroosmotic boundary condition on the sphere surfaces (eq 10):

10 $$V_{s}=\frac{\zeta \epsilon }{\eta }E_{\tan}$$

Where *V*_*s*_ is the slip velocity on the particle surface, ζ is the zeta potential, and *E*_*tan*_ is the is the tangential component of the electric field.

## Supplementary Material

















## Notes

Negatively charged Pt@SiO_2_ particles moved in 1% H_2_O_2_ solution (Video S1) (AVI)
Positively charged Pt@+SiO_2_ particles moved in 1% H_2_O_2_ solution (Video S2) (AVI)
Chemotaxis of negatively charged Pt@SiO_2_ particles in the H_2_O_2_ gradient (Video S3)­(AVI)
Chemotaxis of positively charged Pt@+SiO_2_ particles in the H_2_O_2_ gradient (Video S4)­(AVI)
Rheotaxis of negatively charged Pt@SiO_2_ particles in the H_2_O_2_ flow (Video S5) (AVI)
Rheotaxis of positively charged Pt@ + SiO_2_ particles in the H_2_O_2_ flow (Video S6) (AVI)
The “jumping” behavior of positively charged Pt@+SiO_2_ particles in the H_2_O_2_ flow (Video S7) (AVI)
Additional experimental results and procedures, as well as further characterization data is available online; a scanning electron micrograph of a Pt@SiO_2_ particle (Figure S1); detailed COMSOL simulation results for both neutral self- diffusiophoresis (Figure S2) and ionic self-diffusiophoresis (Figure S3) mechanisms; schematic illustrations of the experimental setups for chemotaxis (Figure S4) and rheotaxis (Figure S5); characterization data for orientation changes during chemotaxis (Figure S6); rheotactic behavior of positively charged particles under different flow rates (Figure S7); time-lapse microscopy images illustrate the ″jumping″ behavior of particles in external flow (Figure S8) (PDF)

## §

Department of Physical Chemistry of Polymers, Max Planck Institute for Polymer Research, Mainz 55128, Germany
