# Supplementary material for: Programming Tactic Behaviors of Active Colloids via Surface Charge
Source: ACS Nano. 2025 Jun 7;19(23):21460–7. doi: 10.1021/acsnano.5c02441 (PMC12177946; doi:10.1021/acsnano.5c02441)
Supplement: Supplementary file 8 [file nn5c02441_si_008.pdf]

Programming Tactic Behaviors of Active Colloids via Surface Charge

Zuyao Xiao,† Priyanka Sharan,†,¶ and Juliane Simmchen*,†,‡

†*Department of Physical Chemistry, Technische Universität Dresden, Dresden 01069, Germany*

‡*Pure and applied chemistry, University of Strathclyde, Glasgow G11XL, UK*

¶*Current address: Department of Physical Chemistry of Polymers, Max Planck Institute for Polymer Research, Mainz 55128, Germany*

E-mail: juliane.simmchen@strath.ac.uk

The Supporting Information includes a scanning electron micrograph of a Pt@SiO_2 particle (Figure S1). Also, detailed COMSOL simulation results for both neutral self-diffusiophoresis (Figure S2) and ionic self-diffusiophoresis (Figure S3) mechanisms for negatively and positively charged particles are presented. Schematic illustrations of the experimental setups for chemotaxis (Figure S4) and rheotaxis (Figure S5) assays are included. Further characterization data are presented for orientation changes during chemotaxis, showing the time evolution of the orientation angle and mean-squared angular displacement under various conditions (Figure S6). The rheotactic behavior of positively charged particles under different flow rates is characterized by their velocity along the flow direction and directional rose plots (Figure S7). Time-lapse microscopy images illustrate the "jumping" behavior of particles in external flow (Figure S8). Additionally, seven supporting videos (Videos S1-S7) are available.

Supplementary Figures

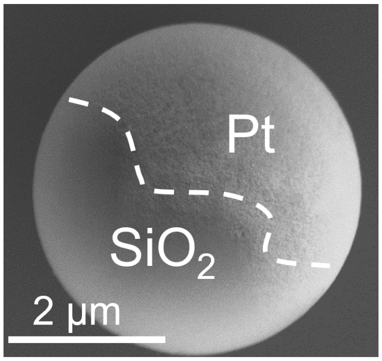

Figure S1: Scanning electron micrograph of Pt@SiO_2.

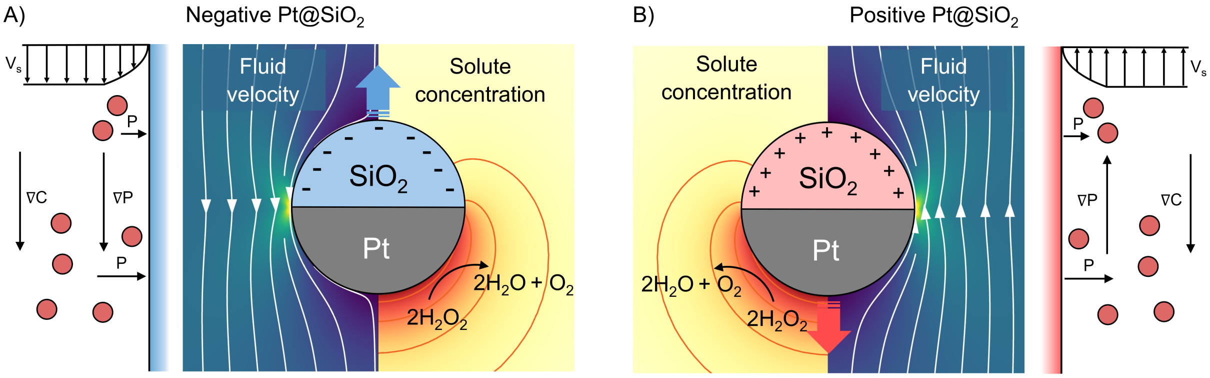

Figure S2: The COMSOL simulation based on neutral self-diffusiophoresis for A) negatively charged Pt@SiO_2 particles and B) positively charged Pt@+SiO_2 particles in H_2O_2. The red circles indicate solute molecules (O_2 here), which are produced from the localized chemical reaction. ∇C represents the tangential component of the solute concentration gradient. P denotes the osmotic pressure generated by the interaction potential between the solute molecule and the particle surface. ∇P denotes the tangential pressure gradient, which drives the slip velocity V_s. For Pt@SiO_2, the repulsive interaction directs V_s toward higher solute concentration, while for Pt@+SiO_2, the attractive interaction causes V_s to be directed toward lower solute concentration. Each contour plot shows the simulated concentration profile for O_2 and the streamline plot shows the simulated fluid flow field. (see Finite Element Simulation for more details)

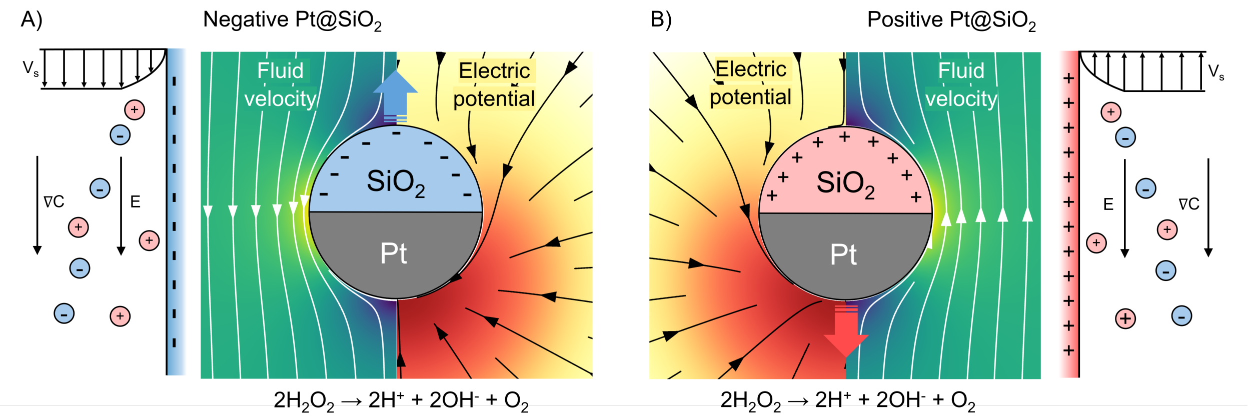

Figure S3: The COMSOL simulation based on ionic self-diffusiophoresis for A) negatively charged Pt@SiO$_2$ particles and B) positively charged Pt@ + SiO$_2$ particles in H_2O_2. The red and blue circles indicate cations (H^+) and anions (OH^-), which are produced from the intermediate species of the decomposition of H_2O_2. ∇C represents the tangential component of the solute concentration gradient. E represents the electric field generated by the difference in ionic diffusivities. V_s is the slip flow at the particle surface driven by the electric field. For Pt@SiO$_2$, the particle surface carries a negative charge ($\zeta < 0$), and the electric field drives fluid flow toward the Pt cap, resulting in propulsion toward the SiO$_2$ side. In contrast, for Pt@+SiO$_2$, where the surface becomes positively charged ($\zeta > 0$) after APTES functionalization, the direction of the induced flow reverses, and the particle propels toward the Pt cap. Each arrow plot shows the simulated electric field generated by ions and the streamline plot shows the simulated fluid flow field. (see Finite Element Simulation for more details)

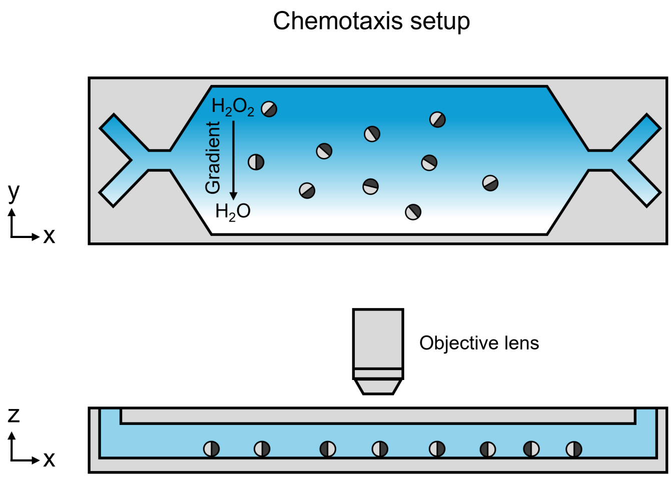

Figure S4: Schematic illustration of the setup of chemotaxis experiments.

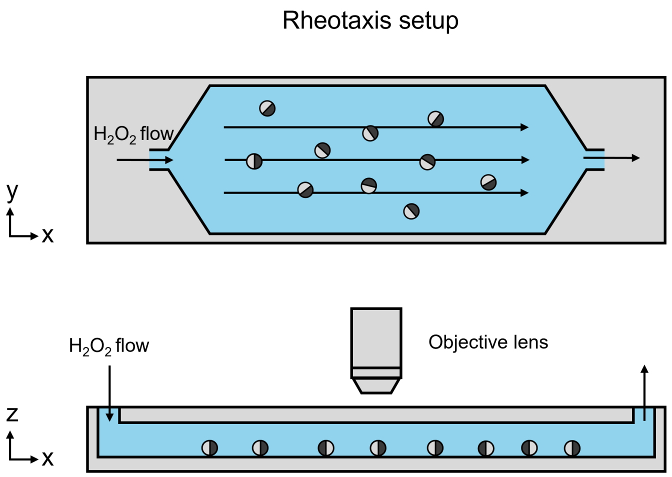

Figure S5: Schematic illustration of the setup of rheotaxis experiments.

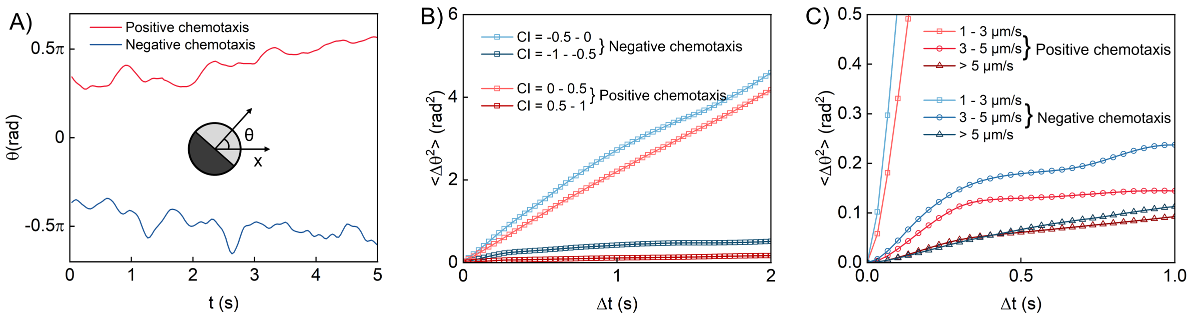

Figure S6: Characterization of orientation changes during chemotaxis. (A)Time evolution of the orientation angle θ for positively charged (red) and negatively charged (blue) Pt@SiO_2 particles under chemotactic conditions. The inset illustrates how θ is defined relative to the x-axis of the observation frame. Over time, the positively charged swimmers converge toward $\theta \approx +\frac{\pi}{2}$, whereas the negatively charged swimmers orient around $\theta \approx -\frac{\pi}{2}$. (B) Mean-squared angular displacement $\langle \Delta\theta^2 \rangle$ versus time, grouped by the chemotactic index (CI). Here, CI is defined as the ratio of a particle's net displacement along (or against) the gradient to its total path length, ranging from -1 (purely negative chemotaxis) to $+1$ (purely positive chemotaxis). Larger $|\text{CI}|$ correlates with smaller angular fluctuations, indicating that strongly chemotactic particles, no matter positive or negative, maintain a more stable orientation. (C) $\langle \Delta\theta^2 \rangle$ grouped by swimming speed, showing that faster-moving particles exhibit smaller angular fluctuations. Higher speeds thus correlate with stronger alignment to (or away from) the gradient and more pronounced chemotaxis.

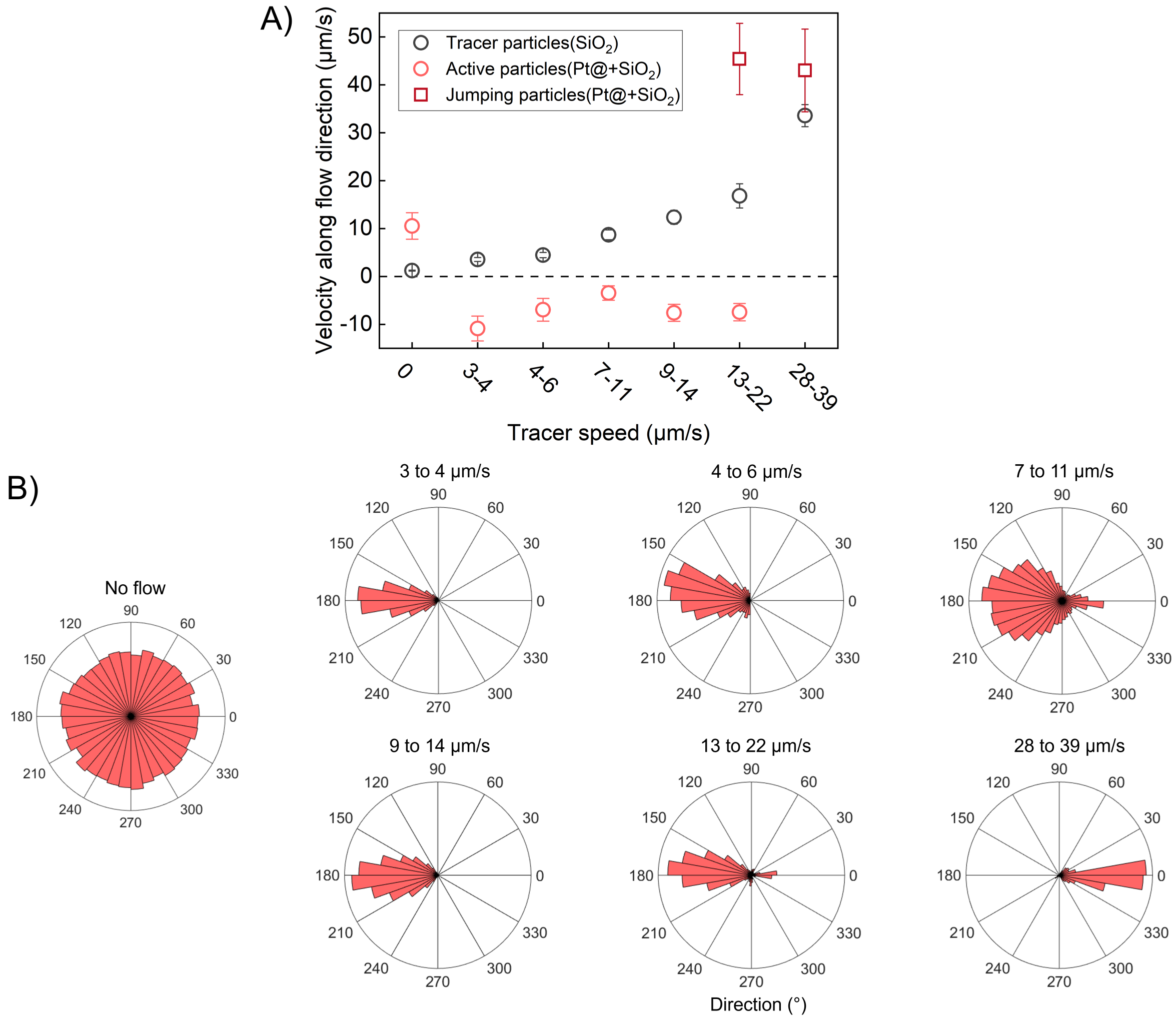

Figure S7: Characterization of rheotaxis for positively charged Pt@SiO_2 under different flow rates. A) Velocity of Pt@SiO_2 particles along the x-axis (the imposed flow direction) compared to SiO_2 tracer particles at various external flow speeds. Positive velocities denote motion in the direction of flow, while negative values signify motion against it. The black circles correspond to tracer particles, the red circles are active Pt@SiO_2, and the red squares represent "jumping" particles observed at higher flow rates. B) Rose plots illustrating the moving direction of Pt@SiO_2 across different flow-speed ranges. As the flow rate increases, particles gradually shift from more isotropic orientations (no-flow case) to upstream motion against the flow, showing "jumping" at the highest flow rates.

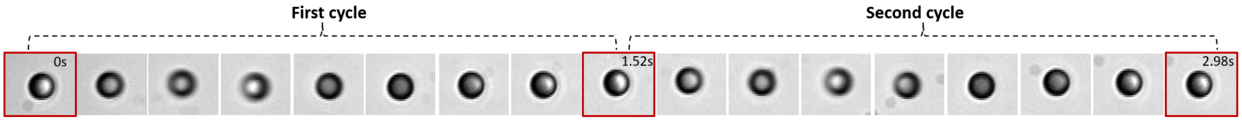

Figure S8: Time-lapse microscopy images illustrating two cycles of the "jumping" behavior in external flow. The particle goes out of focus when it detaches from the substrate.

Supplementary Videos

Supplementary video S1: Negatively charged Pt@SiO_2 particles moved in 1% H_2O_2 solution.

Supplementary video S2: Positively charged Pt@+SiO_2 particles moved in 1% H_2O_2 solution.

Supplementary video S3: Chemotaxis of Negatively charged Pt@SiO_2 particles in the H_2O_2 gradient.

Supplementary video S4: Chemotaxis of Positively charged Pt@+SiO_2 particles in the H_2O_2 gradient.

Supplementary video S5: Rheotaxis of Negatively charged Pt@SiO_2 particles in the H_2O_2 flow.

Supplementary video S6: Rheotaxis of Positively charged Pt@ + SiO_2 particles in the H_2O_2 flow.

Supplementary video S7: The "jumping" behavior of Positively charged Pt@+SiO_2 particles in the H_2O_2 flow.
